# Simultaneous impedance spectroscopy and fluorescence microscopy for the real-time monitoring of the response of cells to drugs†
†Electronic supplementary information (ESI) available: Additional details on the simultaneous setup and mathematical modelling, control experiments on potential dye cytotoxicity, selecting the most sensitive frequency, XPS and electrochemical characterization of the modified surfaces. See DOI: 10.1039/c6sc05159f



**DOI:** 10.1039/c6sc05159f

**Published:** 2017-01-03

**Authors:** M. Parviz, K. Gaus, J. J. Gooding

**Affiliations:** a School of Chemistry , ARC Centre of Excellence in Convergent Bio-Nano Science and Technology , University of New South Wales , New South Wales 2052 , Australia . Email: justin.gooding@unsw.edu.au; b Australian Centre for NanoMedicine , University of New South Wales , New South Wales 2052 , Australia; c EMBL Australia Node in Single Molecule Science , ARC Centre of Excellence in Advanced Molecular Imaging , University of New South Wales , New South Wales 2052 , Australia

## Abstract

A dual fluorescence microscopy and electrochemical strategy to investigate how cell–surface interactions influence the cellular responses to cues for the cell-based biosensing of drug efficacy is reported herein. The combined method can be used to not only monitor the importance of controlling the cellular adhesive environment on the cell response to drugs but it also provides biological information on the timescales of downstream outside-in signaling from soluble cues. As an example of the use of the combined method, we show how adhesive cues influence the signalling responses of cells to soluble cues. G-protein-coupled receptors were used as the target for the soluble cues. The changes in cell adhesion, cell morphology and Ca^2+^ flux induced by soluble histamine were simultaneously monitored as a function of the spacing of the adhesive ligand RGD on the interdigitated indium tin oxide electrodes. The simultaneous measurements revealed that the timescales of histamine-induced Ca^2+^ mobilization and the decrease in cell–cell adhesions are correlated. Furthermore, cells on the surfaces with an RGD spacing of 31 nm were shown to display a faster release of Ca^2+^ and change in cell adhesion upon histamine stimulation compared to cells on other surfaces.

## Abstract 2

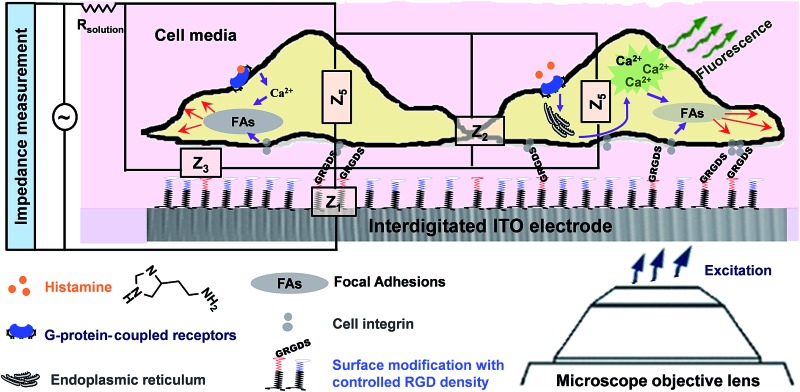
A dual fluorescence microscopy and electrochemical strategy to investigate how cell–surface interactions influence the cellular responses to cues for the cell-based biosensing of drug efficacy is reported herein.

## Introduction

Many environmental cues, including a large number of prescription drugs, target cells through G-protein-coupled receptors (GPCRs) as the largest family of cell–surface receptors.^1,2^ The binding of an external signal to a GPCR typically results in the stimulation of the complex interconnected signaling pathways through which cells coordinate a wide variety of fate decisions. Therefore, there is a tremendous interest in developing cell chip technologies based on monitoring different events on the activity of GPCRs.^3^ The kinetics of these cellular pathways significantly differ from the milliseconds timescale (*e.g.*, GPCR conformational changes or Ca^2+^ flux) to hours (*e.g.*, cytoskeletal modulation).^4^ The GPCRs monitoring technologies are mainly based either on optical measurements^5^ or electrical detections.^6–9^ Among these, fluorescence microscopy^10^ and electrical impedance spectroscopy^11^ are two of the more popular methods, and are, in fact, complementary. Fluorescence microscopy enables the precise monitoring of short-lived specific sub-cellular processes, organelles, and proteins with labeled fluorescent tags.^10^ In contrast, cell-based impedance measurements, which were pioneered by Giaever and Keese,^12^ provide real-time information on the minute changes in the adhesion of cells to cells/surfaces at the whole-cell level over extended periods of time in a label-free manner.

In organisms, cells are surrounded by an extracellular matrix containing adhesive ligands that modulate cell behavior. Hence, using molecularly engineered surfaces with controlled expressions of cell adhesive ligands has started to become more prevalent in the development of cell chips.^13^ It has been demonstrated that a varied spatial distribution of adhesive RGD ligands regulates not only the cell phenotype but also the outside-in and inside-out signalling processes.^14–16^ However, knowledge of how the dynamics of cellular responses to a soluble cue is influenced by surface chemistry is still in its infancy, but is of significant importance in the *in vitro* testing of drugs.

**Fig. 1:**
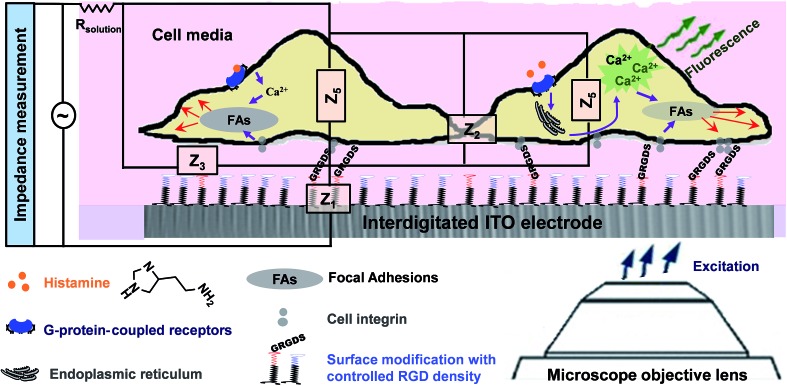
The designed simultaneous set-up for collecting more comprehensive information on cells responses to soluble cues in the presence of tuned adhesive ligands on interdigitated indium tin oxide (ITO) surfaces. Histamine was used as a model soluble cue ligand as it activates G-protein-coupled histamine receptors, and, consequently, Ca^2+^ ions are mobilized from endoplasmic reticulum (ER), followed by cytoskeleton rearrangement through remodelling of the focal adhesions. The presence of Gly-Arg-Gly-Asp-Ser (GRGDS), which act as adhesive ligands to the integrins of cells, also leads to focal adhesion formation and signalling. The impedance results are integrations of the impedance that are attributed to the media–surface interactions (*Z*_1_), cell–cell connections (*Z*_2_), cell–surfaces adhesion (*Z*_3_) and the impedance of cells (*Z*_4_ and *Z*_5_), in addition to the resistance of the solution (*R*_solution_).

In the present study, we sought to combine impedance spectroscopy and live fluorescence microscopy, simultaneously, to provide a platform technology to investigate GPCRs' activity in a more comprehensive manner. This was achieved using optically transparent interdigitated indium tin oxide (ITO) electrodes, which we have shown can be precisely modified with self-assembled monolayers to given biointerfaces with a controlled presentation of cell adhesive ligands. Fluorescence microscopy was utilized to monitor transient histamine-induced Ca^2+^ release from the endoplasmic reticulum (ER). Impedance spectroscopy was employed to acquire information on the dynamic changes in cell adhesion, which can be regulated by both surface chemistry and the soluble GPCRs stimulators. Coupling the fluorescence and impedance readout methods is particularly relevant for tracking events with significantly different timescales but that are connected through GPCRs, as this would not be possible using a single detection method. Furthermore, application of the developed technique can be extended to investigate the possible effects of surface designs on cellular events in response to soluble cues and their timescales. The summarized measurement principles of the work are schematically illustrated in Fig. 1.

## Experimental methods

### Chemicals

16-Phosphohexadecanoic acid (PHDA) of 99.5% purity, dimethylaminopropylcarbodiimide (EDC), *N*-hydroxysuccinimide (NHS), dichloromethane, *N*,*N*′-disuccinimidyl carbonate (DSC), 4-(dimethylamino)pyridine (DMAP), acetonitrile and anhydrous dimethyl sulfoxide (DMSO) were purchased from Sigma-Aldrich (Sydney, Australia). 1-Aminohexa(ethylene oxide) and 1-aminohexa(ethylene oxide) monomethyl ether were obtained from Biomatrik Inc (Jiaxing, China). Dulbecco modified Eagle medium (DMEM), l-glutamine and fura-2 AM were purchased from Invitrogen (Sydney, Australia). Gly-Arg-Gly-Asp-Ser (GRGDS) was obtained from Genscript (Sydney, Australia).

### Cell culture

HeLa cells (CCL-2, American Type Culture Collection) were grown to confluence in DMEM culture media supplemented with 10% fetal bovine serum and 0.1% glutamine at 37 °C in a humidified atmosphere with 5% CO_2_. Every 3–4 days, the cells were detached from the culture flask using trypsin and washed with PBS containing 0.9% sodium chloride, and were then resuspended in fresh media. The suspended cells were counted, and a high density of 1.5 × 10^5^ cells per cm^2^ in 650 μL of the media was seeded on each modified interdigitated ITO surface for 3.5 h or until an 80-98% confluent cell layer is reached on bare surfaces.

### Loading HeLa cells with fura-2 AM

The plated HeLa cells on interdigitated ITO surfaces were washed twice using phenol red-free DMEM before being loaded with fura-2 AM dyes. The loading was performed using the protocol developed by Herman's group.^17^ Briefly, the cells were incubated in phenol red-free DMEM containing 10% fetal bovine serum (FBS), 2 μM fura-2 AM and 1 μL of 10% w/v Pluronic F-127 in DMSO for 45 min at room temperature. The cell-covered surfaces were washed twice with an imaging buffer made of Hanks' balanced salt solution plus 0.8 mM MgCl_2_, 1.6 mM CaCl_2_ and 5% FBS.

### Preparation and addition of histamine solution

Histamine solution was prepared freshly by dissolving the histamine powder in Hanks' balanced salt solution. In each experiment, 50 μL of the 1.5 mM histamine solution was pipetted in to the media. The final concentration of histamine in the media was 100 μM. In addition, before adding histamine, 50 μL of Hanks' balanced salt solution was pipetted to the exciting cell media in the chamber to indicate that there was no change by adding just the buffer. The addition of the solutions was done through the hole in the chamber lid.

### Simultaneous live microscopy and real-time impedance measurements

The interdigitated ITO electrodes used in this study consisted of 65 pairs of finger electrodes 10 μm wide with a 5 μm spacing between the electrodes, which were 2 mm in length and approximately 100 nm thick on rectangular AT-cut quartz crystals approximately 0.45 mm thick. The electrodes were placed inside the custom-made electro/optical chamber (Micrux technologies, Oviedo, Spain) and put under the microscope while it was connected to the potentiostat (SP 200, BioLogic Science Instruments). For the impedance measurement and analysis, the interdigitated ITO surfaces were probed with a weak non-invasive AC signal within the frequency range of 400–40 000 Hz (with 10 mV AC voltage). The impedance of the modified cell-free interdigitated ITO electrodes was measured as the baseline data. Then, the real-time impedance readout of the cell layer 10 min before and continuously 3 h after the histamine addition was monitored. The three-electrode system measures the signals from one half of the interdigitated pairs of electrodes, that is from 65 ITO microelectrode fingers, with an active surface area of around 1.3 × 10^–2^ cm^2^.

Histamine was added after the surfaces containing cells were equilibrated at 37 °C and the impedance level stabilized. The impedance values were normalized by dividing the impedance of the cell-covered electrode to the baseline impedance. EC-Lab® V10.33 (BioLogic, Science Instruments) software was used to analyze the impedance data. The sequences of the fluorescence and phase-contrast images were acquired on a Zeiss Axio Observer X.1 Spinning Disc/TIRF inverted microscope with Zeiss ZEN software. The microscope was equipped with a lambda DG4 light source switcher and a scientific complementary metal-oxide semiconductor (sCMOS) camera (Orca Flash 4). The wavelength switcher allowed shifting between two wavelengths in less than 1.2 ms.

### Ratiometric analysis

The herein reported average ratiometric values are the mean of the ratiometric value of cells in the field of view. The ratiometric value at each time point was calculated using Fiji software as follows: by opening the movie recorded from ensembles of cells > choosing split channels > extracting the frame of the time of interest > drawing the rough area around each individual cells, regions of interest (ROI), using the “freehand selection” tool > saving these areas using “ROI manager” > measuring the mean grey value of each ROI of each channel > subtracting the background intensity > dividing the aligned emission intensity excited at 340 nm by that at 380 nm, pixel by pixel, over each manually traced cell region after subtracting the background fluorescence intensity. The background fluorescence intensity was obtained by calculating the mean grey value of a cell-free area on each frame. The ratiometric value of each individual cell was normalized by dividing the fura-2 ratio value with the subtracted background data at each time point with the average ratiometric value of that cell during the last 2 min before histamine addition, again after subtracting the background value.

The time-resolution ratiometric images of an individual cell were obtained from the created ratiometric movie of the ensemble of cells as follows; by drawing the ROI around the cells in the ratiometric movie > choosing Image > Stacks > Tools > Make Substacks. Then, the ratiometric movie of the single cell was automatically created by the software. The frames related to the time point of interest were extracted from the movie. The false colour images were created using the “Lookup Tables” in the “Image” tab of the Fiji software. The summarized average variations in the percentages of the fura-2 ratio were calculated by subtracting the average ratiometric values at the beginning of the experiment from the maximal ratiometric values after the histamine addition.^18^

### Analysis of the impedance of the cell layer

In this study, these values were calculated based on fitting the mathematical model to the experimentally-acquired frequency-resolved impedance raw data using MATLAB® software from The MathWorks Inc (Natick, MA. Pelli, DG), using the fitting curve function.

### Preparation of the RGD-controlled surfaces

A multi-step strategy was followed to modify the surfaces chemically with RGD molecules with defined spacing. To this end, the interdigitated ITO surfaces were first rinsed with copious amounts of Milli-Q water and methanol and were then placed in an oxygen-plasma cleaner after being dried under a nitrogen stream (Harrick Plasma Cleaner/Sterilizer PDC-32G, Ossining, NY) for 5 min. PHDA SAMs were assembled from 400 μL of a 5 mM PHDA solution in methanol for 12 h. The surfaces were next rinsed with methanol and Milli-Q water and annealed at 150 °C for 48 h under vacuum. To further modify the PHDA SAM, the terminal carboxylic acid groups were activated with 400 μL of 50 mM EDC/NHS in an aqueous solution for 2 h. The succinimide ester activated groups of the PHDA SAMs then were incubated in a 200 μL solution containing various ratios of 1-aminohexa(ethylene oxide) to 1-aminohexa(ethylene oxide) monomethyl ether for 12 h to bond to their amine groups. These molecules provide the inert background to avoid nonspecific cell adhesion.^19^ The ratio of these two ethylene oxide molecules is the key to regulating the RGD spacing on the surfaces. The modified surfaces were rinsed with DMSO several times and then dried under a nitrogen stream. The hydroxyl-terminated 1-aminohexa(ethylene oxide) molecules were then activated with 400 μL of 0.1 M DSC/DMAP in anhydrous DMSO for 6 h. The activated surfaces were washed with a copious amount of DMSO and then Milli-Q water, followed by immersion in 400 μL 20 mM phosphate buffer containing 15 μg mL^–1^ of GRGDS for 30 min at room temperature. GRGDS attaches to hexa(ethylene oxide) molecules on the surface by forming amide bonds.^20,21^ The modified interdigitated ITO surfaces were washed with Milli-Q water and PBS three times for 5 min each time to remove any physically adsorbed peptides. Solvent evaporation was prevented during the surface modification by incubating the samples in a glass container with a sealed lid. Small open petri dishes, containing additional solvent, were also placed inside this sealed container to keep the environment vapour rich. Finally, the surfaces were sterilized with 70% ethanol and then rinsed with PBS in a safety cabinet before plating the cells.

### Characterization of the RGD-controlled surfaces

XPS analyses were conducted using an ESCALAB 220iXL spectrometer equipped with a monochromatic Al Kα source (1486.6 eV), a hemispherical analyzer and a multichannel detector. The spectra were obtained in normal emission with the analyzing chamber operating below 10^–10^ mbar. The angle of incidence with respect to the analyzer lens was set to 58° with a spot size of approximately 1 mm^2^. High-resolution spectra and wide surveys were obtained using a pass energy of 20 and 100 eV, respectively. The experimental energy shifts were corrected with reference to C 1s at 284.8 eV. The fitting of the spectra was performed using a nonlinear least-squares procedure and a simple Lorentzian–Gaussian function. The percentage coverage for the different elements and sub-groups were estimated from the respectively fitted area and the attributed sensitivity factors. To further characterize the modified surfaces, cyclic voltammograms before and after modification of an interdigitated ITO electrode with the self-assembled PHDA and GRGDS were recorded. The XPS results were used to estimate the RGD spacing (see the ESI for more detail†) of the prepared surfaces, as we have reported previously.^22^

### Statistical analysis

Herein, the data are displayed as means ± RSD unless otherwise stated. Data were analyzed using the GraphPad Prism version 6.0 data management software to conduct ANOVA on groups of data. For statistics, the minimum number of repeated experiments “*n*” was equal to 3.

## Results

**Fig. 2:**
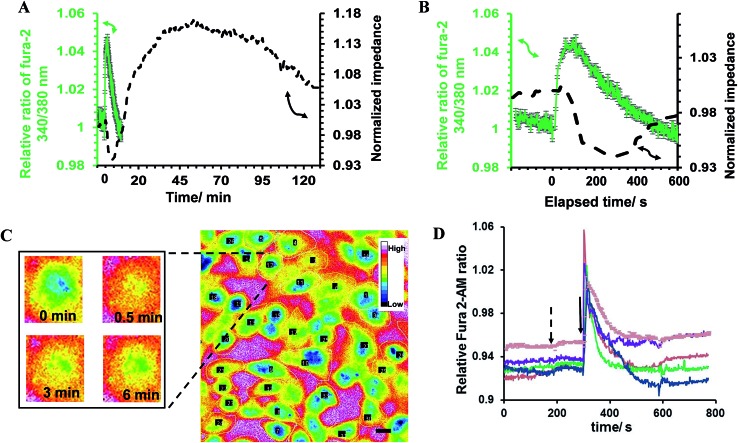
(A) Relative average 100 μM histamine-induced change in the fura-2 ratio at 340/380 nm indicating the release of Ca^2+^ and the simultaneously recorded impedance of HeLa cells plated on a bare interdigitated ITO electrode. Cells were cultured on such surfaces until reaching an 80–98% confluent cell layer. The normalized impedance was calculated by dividing the impedance of the cell-covered electrode by the impedance of the cell-free one. (B) Expansion of the impedance and Ca^2+^ mobilization response for short time intervals from part (A) showing the correlation between the timescale of the fluorescence spike and the decline in the impedance below baseline levels. (C) Representative of an ensemble of fura 2-loaded HeLa cells plated on the surface. The time-resolved images of one of the cells are shown as an example. Images are pseudocoloured with warmer colours indicating a higher ratiometric fluorescence intensity in the cell regions. The respective elapsed time after histamine addition is shown in the images. (D) Each curve presents the change for one single cell. The ratiometric values of some cells in the field of view are shown in part (A) as examples of the data used to calculate the average change in the ratiometric value. All the fura-2 loaded cells were responsive to 100 μM histamine stimulation. The arrows indicate the time of Hanks' balanced salt solution (dashed arrow) or histamine (solid arrow) addition. Scale bar is 20 μm.

In the first part of the study, the feasibility of combining both impedance and live fluorescence measurements on interdigitated ITO surfaces was examined. A custom-made electro/optical chamber was employed to provide the possibility of running electrical measurements and inverted microscopy on transparent interdigitated ITO electrodes (a picture of the simultaneous measurement set-up can be found in the ESI (Fig. S1)).† The experimental conditions were kept ideal for live cells by controlling the temperature at 37 °C and maintaining the atmosphere humid with 5% CO_2_ in the microscope cage. The response of the fura-2-loaded HeLa cells on the interdigitated ITO surfaces to 100 μM histamine was explored using the developed set-up. The simultaneously recorded histamine-induced impedance changes and Ca^2+^ flux are shown in Fig. 2A. The average Ca^2+^ peak reached a maximum within 60 s and then decreased to a baseline level in a further 490 s. At the same time, histamine caused a rapid dip in impedance that persisted for a few minutes before restoring to the initial value. Thereafter, the impedance increased over a time span of approximately 35 min, before returning to a basal level after a further 80 min. The real-time impedance results are reported at the frequency of 4000 Hz. We found this frequency as the most sensitive frequency to display impedance alteration caused by the HeLa cell layer among the examined frequencies between 40 000 Hz and 400 Hz (Fig. S2 shows some examples (ESI)†). Notably, the observed trend for the changes in impedance and calcium ion mobilization are in agreement with those in previous studies employing impedance spectroscopy on gold^23^ or Ca^2+^ flux measurements on glass coverslips.^17^ Thus, we demonstrated that the developed set-up allows the monitoring of sub-cellular and whole-cell responses of ensembles of cells over a wide timescale from milliseconds to hours. Fig. 2C presents the image of an ensemble of fura-2-loaded HeLa cells and time-lapse montages of one of the cells responding to histamine, as an example. These images show that high-quality fluorescence images are obtainable from the surfaces using the present set-up. Examples of the fura-2 ratio alteration of some individual cells in the cell layer in the field of view over time are shown in Fig. 2D. The response of many more cells can be found in Fig. S3 (ESI†). The results indicate that the concentration of 100 μM of histamine evokes the cytosolic Ca^2+^ release by almost all the fura-2-loaded cells. This is the reason for choosing this concentration, as it has also been reported previously.^24^ Here, histamine was dissolved in Hanks' balanced salt solution. It was seen that the addition of Hanks' balanced salt solution alone caused no significant changes in calcium signals. The addition of buffer also did not cause any change in the impedance values (results not shown here). These results indicate that the obtained responses purely originated from the histamine stimulation. Furthermore, we investigated whether the fura-2 loading and imaging process for the time window of the fluorescence recording interrupted the impedance results of the cells by recording the cell responses using impedance spectroscopy and phase-contrast microscopy on dye-free HeLa cells, as shown in Fig. S4 (ESI†). The simultaneous measurement revealed that the onset of the impedance alteration was delayed by 30–60 s after the intracellular Ca^2+^ changes. In addition, it was seen that the timescale of the fluorescence response from the Ca^2+^ mobilization was correlated with the time of the dip in the impedance signal before it was restored to the initial value (Fig. 2B).

The impedance results were further analyzed to obtain the contribution the changes in the cell–cell and cell–surface adhesion make to the alteration of the impedance value.^12,25,26^ To this end, frequency-resolved impedance readings (40 000–400 Hz) at different time points were subjected to a mathematical model developed by Giaever and Keese^12^ (Fig. S5 (ESI†)). In this model, the current is assumed to flow radially into the space between the cell's ventral surface and the substrate and then escapes between the cells. The current density is assumed to be consistent in the *z*-direction and the cells are approximated as disc-shaped objects with insulating membrane surfaces and filled with a conducting electrolyte.^12^ The total impedance of the monolayer is made up of the impedance between the ventral surface of the cell membrane and the substratum, the impedance between the cells and the impedance of the cell itself, which is dominated by the membrane capacitance. According to this model, the specific impedance for a cell-covered electrode can be written according to the following equation:^12,25^ 
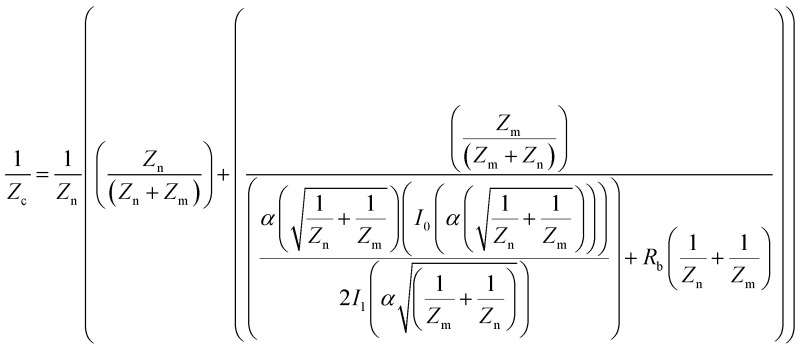
where *I*_0_ and *I*_1_ are modified Bessel functions of the first kinds of order 0 and 1, respectively; *Z*_n_ (Ω cm^2^) is the impedance associated with the electrode/electrolyte interface, measured as the impedance of the cell-free electrode at different frequencies; and *Z*_c_ (Ω cm^2^) is the specific impedance for the cell-covered electrode. Here, the values of *Z*_c_ and *Z*_n_ were measured experimentally at 12 different frequencies (*f*) spaced evenly on the logarithmic scale ranging from 40 000 Hz to 400 Hz (10 mV). Also, in this equation, *α* and *R*_b_ describe the contributions cell–surface and cell–cell adhesion make to defining the total cell-covered impedance, respectively. Therefore, the values of *α*, *R*_b_ and *Z*_m_ are the only adjustable parameters that can be obtained by finding the best fit of the impedance data.^27^ The resistance of the media was subtracted from the measured impedance before the calculations were done and were then added back for comparing the experimental and calculated data. *Z*_m_ is dominated by the cell-specific membrane capacitance (*C*_m_) according to the following equation:*Z*_m_ = 1/2π*fC*_m_

**Fig. 3:**
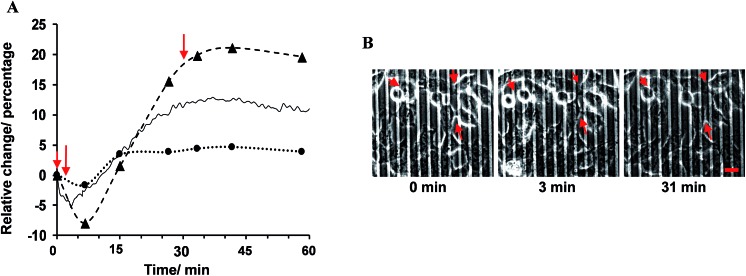
(A) The experimental frequency-resolved impedance spectroscopy was fitted to the mathematical equation to estimate the contribution the changes in cell–cell connectivity (dashed line) and cell–substrate adhesion (dotted line) make in altering the overall impedance response. These data are shown along with the experimental impedance at 4 kHz (solid line). This frequency was detected to be the most sensitive frequency in revealing the impedance change in response to histamine addition in the examined range. (B) Representative time-lapse montages of HeLa cells before the addition of histamine, and at 3 min and 30 min after histamine stimulation, captured using phase-contrast microscopy performed simultaneously with impedance measurements, as shown in part (A). The arrows in part (A) indicate the times that the phase-contrast images were taken from the recorded video. The arrows on the images point to the locations with observable changes in the cell–cell distance. Scale bar is 20 μm.

The percentage change in *α*, *R*_b_ and the experimental data with respect to their values before the addition of histamine is expressed herein as the relative change, as shown in Fig. 3A. The results showed that the observed immediate decrease in the experimental impedance was mainly caused by the decrease in cell–cell resistance (*t* < 4 min). Here, the active surface area of the interdigitated ITO area was 1.3 × 10^–2^ cm^2^. The impedance value calculated from the mathematical model showed that the average *R*_b_ value changed from 1.80 ± 0.10 to 2.13 ± 0.12 Ω cm^2^, while the average *α* value was altered from 1.68 ± 0.04 to 1.72 ± 0.05 Ω^1/2^ cm in 25 min after histamine addition compared to the respective values before histamine addition. The *α* values can be used to extract the morphological cell parameters by approximating the cells as disc-shaped objects according to the following equation:^12^ *A* = *r*_c_√(*ρ*/*h*)where *r*_c_ (μm) is the average cell radius, *ρ* (Ω cm) is the specific resistivity of the cell culture medium and *h* (Å) is the average height between the ventral cell membrane and the surface. The value of *h* displays the integration of both the focal and non-focal adhesions in defining the distance between the cell surface and the substratum. In the current study, no significant change in cell radius was observable using microscopy observation. By setting the average cell radius of HeLa cells to 10.2 ± 2.1 μm based on microscopy images and *ρ* to 54 Ω cm at 37 °C, the value of *h* was calculated to change from 199.5 ± 82.1 to 190.0 ± 78.6 nm after 25 min of exposure to histamine. In addition, the *C*_m_ values changed from 1.52 ± 0.13 μF cm^–2^ before histamine addition to 1.43 ± 0.16 μF cm^–2^ after histamine stimulation. The observed changes do not represent significant alterations in the cell membrane capacitance or cell–surface distance. These findings are in agreement with studies on endothelial cells suggesting that histamine targets E-cadherins in endothelial cells, and dominantly decreases cell–cell connectivity.^28^ Cadherins are Ca^2+^-dependent transmembrane proteins. Phase-contrast images of cells were recorded before and after histamine addition in an attempt to visualize the change in cell–cell distances using the present system set-up (Fig. 3B). The arrows on the images show the locations where the short-term increase and long-term decrease in cell–cell distances in response to histamine can be observed. It is worth noting that the quality of the presented phase-contrast images is compromised by the thickness of the slides employed and is not an inherent issue of ITO surfaces. An example of a high-quality image from ITO surfaces is presented in Fig. S6 (ESI†). This indicates that, in addition to live fluorescence microscopy, the system can be optimized to perform phase-contrast microscopy simultaneously with impedance signal measurement.

**Fig. 4:**
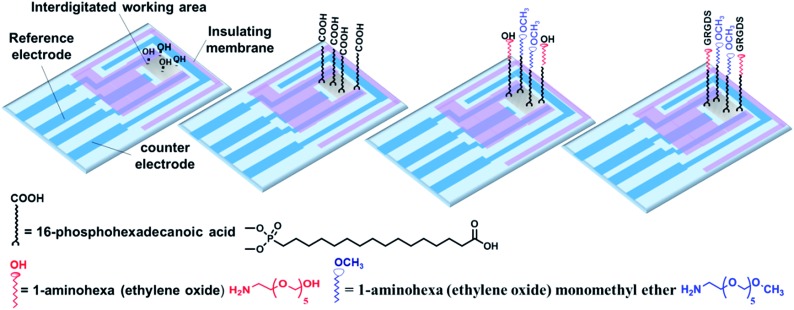
Interdigitated ITO area modified using 16-phosphohexadecanoic acid, followed by attaching to various ratios of 1-aminohexa(ethylene oxide) to 1-aminohexa(ethylene oxide) monomethyl ether. Further coupling of a controlled amount of GRGDS cell adhesive ligands was achieved with the hydroxyl-terminated, but not to the methoxy-terminated, species.

The ability to screen live cells in a physiologically relevant context is crucial to acquiring reliable information for drug discovery and development. Therefore, surface chemists have been motivated to engineer substrates to provide a defined expression of immobilized ligands.^29^ To date, seldom has the effect of surface chemistry been connected with the biological functions of cellular targets in response to soluble cues in real-time. Therefore, in the present study, interdigitated ITO surfaces were modified with various RGD spacings to assess any possible impact of cell adhesive ligand expression on soluble drug-induced cellular responses (Fig. 4). The modification strategy and characterization of the surface are outlined in detail in the Methods section and in the ESI (Fig S7 and S8).† In brief, a plasma-cleaned interdigitated ITO surface was modified with 16-phosphohexadecanoic acid self-assembled monolayers, followed by coupling it to different ratios of 1-aminohexa(ethylene oxide) to 1-aminohexa(ethylene oxide) monomethyl ether molecules. The distal hydroxyl on the 1-aminohexa(ethylene oxide) was then activated and the GRGDS adhesive ligands attached. The RGD spacing on the surfaces was tuned by adjusting the ratio of the two ethylene oxide-based molecules to form a wide range of nanometre to micrometre average RGD spacings on the surfaces. Based on XPS analyses, the GRGDS spacing was estimated to be around 1 nm for the surfaces modified with only 1-aminohexa(ethylene oxide), 41 nm for a ratio of 1 : 10^3^, 970 nm for 1 : 10^6^ and 31 000 nm for a ratio of 1 : 10^9^ of 1-aminohexa(ethylene oxide) to 1-aminohexa(ethylene oxide) monomethyl ether. The high density of cells on these surfaces were stimulated with 100 μM histamine as the model drug after 3.5 h culturing.

**Fig. 5:**
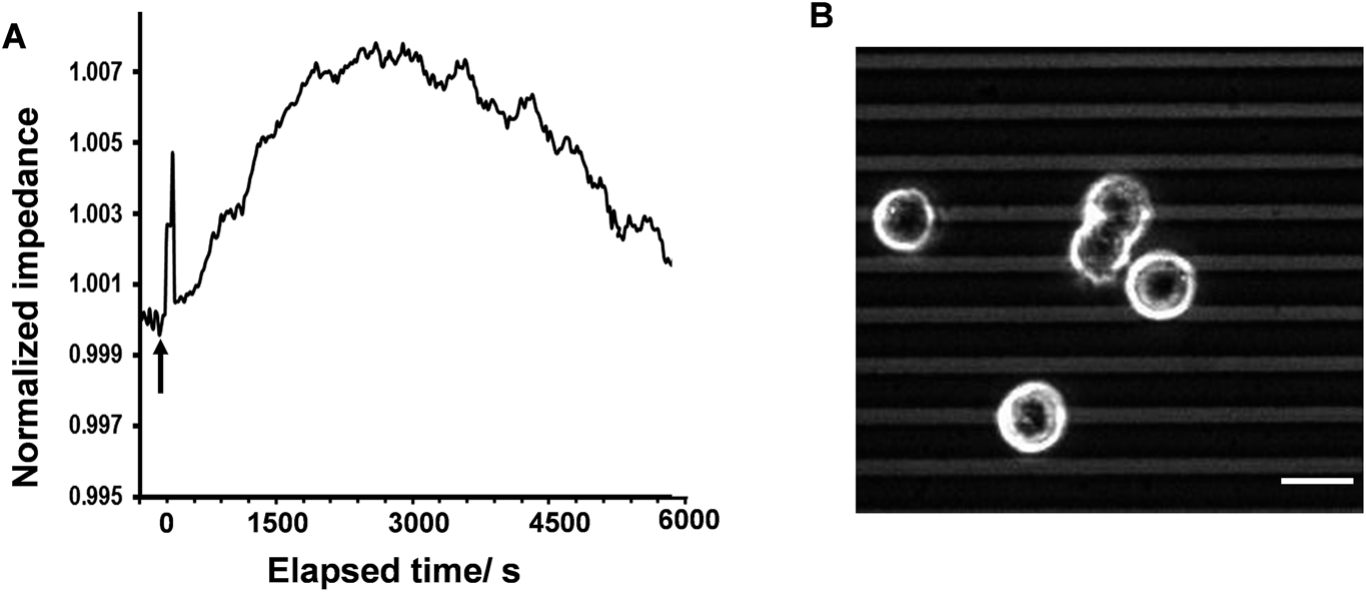
(A) Histamine-induced impedance alteration of cells on the surface modified with only 1-aminohexa(ethylene oxide) monomethyl ether molecules (no RGD) after 3.5 h of plating the cells and before the fura-2 dye-loading procedure. This peak displayed a weakly visible change (less than 1%), with no initial immediate decrease on this surface. (B) Phase-contrast image of cells on this surface. These data further support the idea that a reduction in cell–cell adhesion precedes the immediate histamine-induced decrease in the impedance value.

Cells on surfaces modified with 1-aminohexa(ethylene oxide) monomethyl ether in the absence of 1-aminohexa(ethylene oxide), such that no RGD ligands were attached, did not show any reproducible histamine-induced Ca^2+^ response. Cells on this surface displayed a slight change (less than 1%) in the impedance signal, with no initial immediate decrease upon histamine stimulation (Fig. 5A). The recorded phase-contrast images on this interface indicate that only a limited number of cells were adhered to the surface, and rarely any mature cell–cell contact formed (Fig. 5B). These results further support the conclusions from the fitting of the impedance responses that a decrease in cell–cell adhesion dominates the initial histamine-induced decrease in impedance.

**Fig. 6:**
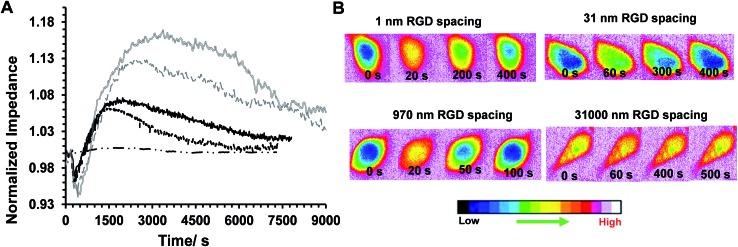
(A) Some examples of simultaneously recorded real-time normalized histamine-induced impedance on surfaces with various RGD spacings of: 1 nm (black solid line), 31 nm (black dashed line), 970 nm (grey solid line) and 31 000 nm (grey dashed line), and the surface with no RGD (black dash-dotted line). (B) Some examples of simultaneously recorded time-lapse montages of fura-2-loaded single HeLa cells on surfaces with different average RGD spacings (the spacing is shown on top of each series of images). The elapsed time written on each image refers to the duration after histamine addition. The pseudocolour calibration bar indicates that warmer colours (*e.g.* orange, red) can be attributed to the regions with a higher concentration of intracellular calcium cells.

**Fig. 7:**
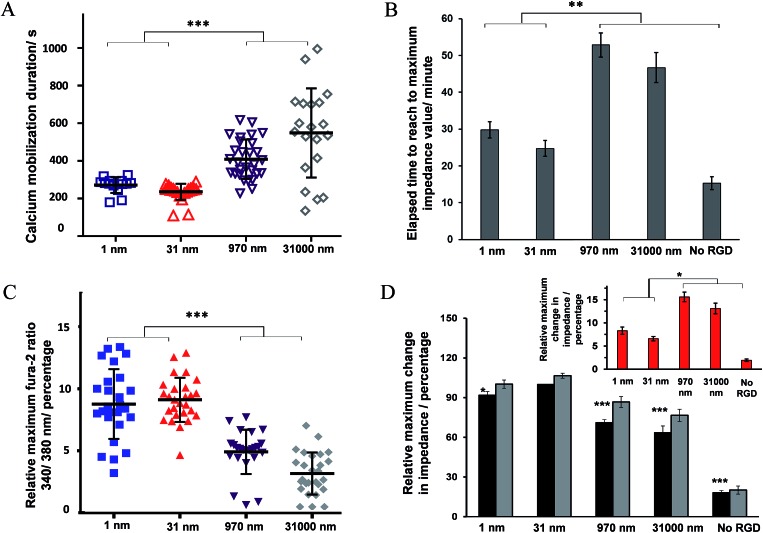
(A) Ca^2+^ mobilization duration. (B) Time to reach the maximal impedance response. (C) The relative maximum normalized fura-2 ratiometric values. These data were obtained by dividing the fluorescence intensity measured with excitation at 340 nm by that measured at 380 nm for cells. The data were integrated over the ratiometric data of the individual cells in the ensemble of cells at the time of the maximum response divided by the respective ratiometric value of each cell before histamine addition, *n* ≥ 19. (D) Values of the maximum impedance response of cells before (black bars) and after (grey bars) histamine addition normalized to the impedance values of cell-free electrodes. The inset is the difference between the impedance values before and after histamine addition. The results quantitatively show the impact of presenting diverse average RGD spacings to cells on histamine-induced impedance changes and calcium ion release responses to the model drug. For statistics, 1 way ANOVA was run, comparing all the data sets with data of the surface with an average RGD spacing of 31 nm. *p < 0.05 relative to surfaces with 1 nm and 31 nm average RGD spacings, *n* ≥ 3.

Some examples of the Ca^2+^ response and impedance signals of the cells on surfaces with different RGD densities are shown in Fig. 6. Simultaneous measurements revealed that the histamine stimulation of cells on the surfaces with an average RGD spacing of 1 or 31 nm led to cytosolic Ca^2+^ release and impedance changes over shorter durations of time compared with cells on the surfaces with a 970 or 31 000 nm average ligand spatial distribution (Fig. 6B and 7A). The duration of Ca^2+^ was calculated based on the time that the Ca^2+^ signal takes to reach a plateau. In addition, the results indicated that the magnitude of the histamine-induced Ca^2+^ flux (Fig. 7C) and the impedance response (Fig. 7D) were also sensitive to the spatial expression of the RGD adhesive ligands. Histamine promoted a higher Ca^2+^ response in cells plated on the surfaces with an RGD spacing of 1 or 31 nm than on the surfaces with an average RGD ligand spacing of 970 or 31 000 nm.

The inset plot in Fig. 7D shows, for cells on surfaces with an average ligand spacing of 1 or 31 nm, there was a lower relative increase in impedance values upon histamine addition compared with cells on surfaces with an average RGD spacing of 970 or 31 000 nm. These smaller impedance increases could be attributed to the more robust cell adhesion to these surfaces and to neighbouring cells before stimulation than the surfaces with larger ligand coverage. The higher baseline impedance level of the plated cells confirmed the greater cell adhesion to the surfaces with a 1 or 31 nm average ligand spacing compared with the surfaces with a 970 or 31 000 nm average ligand spatial distribution (Fig. 7D, black bars). This is physiologically relevant as an RGD spacing smaller than 70 nm mimics the similar periodic spacing of RGD to that found in fibronectin and collagen, which provide strong cell adhesion points.^16^ The change in the cell–cell and cell–surface adhesions on histamine addition at different time points on these surfaces is shown in Fig. S9 (ESI†). Note, the timescales of calcium release and the initial decrease in cell–cell adhesion remain consistent, even on surfaces with a different expression of RGD ligands.

## Discussion and conclusion

Overall, the results confirmed the sensitivity of inside-out and outside-in cell signalling in response to model soluble cues to the chemistry of the surface. We speculate that among the possible factors that may participate in regulating agonist-induced Ca^2+^ mobilization, the morphological state of cells plays an essential role. This is consistent with the findings that show the addition of cytoskeleton disruptors, such as cytochalasin D, contributes to modulating the release of Ca^2+^ through the formation of signalling complexes that alter the efficiency of the relevant signalling transductions.^30,31^ We have previously demonstrated that cell morphology is regulated on ITO surfaces with varying the RGD spacing.^22^ Such biological processes, however, are incredibly complicated as the cytoskeleton and Ca^2+^ mobilization cross-talk in a bi-directional manner. It has been shown that the rearrangement of cellular actin is Ca^2+^ dependent.^32^ On the other hand, these rearrangements are essential for inducing an impedance change. Therefore, the modulation of the Ca^2+^ elevation of cells, and its timescale, on different surface chemistries could be the reason behind the direct relationship between the time of the calcium-induced fluorescence signal and the duration of the initial decrease in impedance on surfaces with various cell adhesive ligand densities. The duration of the initial decrease in impedance followed by the return to baseline levels upon the addition of histamine was found to correlate well with the duration of Ca^2+^ elevation on interdigitated ITO surfaces with various RGD spacings. For instance, the surface with an average RGD spacing of 31 nm, which had the shortest fluorescence spike upon histamine addition, showed the most rapid impedance increase and the shortest duration of the initial impedance dip. These results are consistent with the known consequences of outside-in signalling pathways. Recently, we demonstrated^15^ that endothelial cells on monolayer-modified silicon surfaces with an average RGD spacing of 44 nm display highly ordered focal adhesions and the greatest signal transduction efficiency. That study is particularly relevant to the results presented herein as it is related to cell adhesion and because it showed that a greater outside-in signal transduction resulted in cells migrating more rapidly on those surfaces than on surfaces with other ligand densities upon stimulation with a vascular endothelial growth factor. The relevance with that report is that it showed that for cells to migrate they must reorganize their actin cytoskeleton, as they must here for the cells to spread and alter the impedance. Therefore, in the present study, the outside-in signalling as a result of histamine stimulation causes the calcium mobilization and an immediate decrease in cell–cell contacts due to disruption of the actin. This loss of cell–cell contacts results in an initial decline in impedance, which then appears to allow the cells to spread and the impedance to increase due to the formation of new cell–cell/surface contacts. The direct correlation between the fluorescence signals and this initial decline in impedance on surfaces that express different RGD densities shows that this combined microscopy/impedance approach allows the timescale of these events to be reliably determined. Therefore the set-up detailed herein can be used to provide biological information on correlation of the timescale of various intracellular events and changes in the cell cytoskeleton rearrangement. From a technical perspective, the study shows the importance of controlling the surface chemistry to avoid the risk of misleading information masking the true cellular responses during drug testing. Developing screening tools similar to the simultaneous methodology that is presented herein provides the ability for researchers to carry out the real-time analysis of cellular responses at sub-cellular and whole-cell levels on surfaces designed to provide a well-controlled environment.

In summary, we developed a novel approach that combines two powerful techniques for examining cell responses: live fluorescence microscopy and impedance spectroscopy, simultaneously on surfaces with controlled chemistry. This coupling offers a more in-depth view of cellular responses to soluble cues in the presence of the physical attributes of adhesive cues with different timescales. This methodology allows a more comprehensive and reliable evaluation of drugs *in vitro*.

## References

[cit1] Venkatakrishnan A., Deupi X., Lebon G., Tate C. G., Schertler G. F., Babu M. M. (2013). Nature.

[cit2] Zambrowicz B. P., Sands A. T. (2003). Nat. Rev. Drug Discovery.

[cit3] George S. R., O'Dowd B. F., Lee S. P. (2002). Nat. Rev. Drug Discovery.

[cit4] Fang Y., Frutos A. G., Verklereen R. (2008). Comb. Chem. High Throughput Screening.

[cit5] Cooper M. A. (2002). Nat. Rev. Drug Discovery.

[cit6] Xi B., Yu N., Wang X., Xu X., Abassi Y. (2008). Biotechnol. J..

[cit7] Yu N., Atienza J. M., Bernard J., Blanc S., Zhu J., Wang X., Xu X., Abassi Y. A. (2006). Anal. Chem..

[cit8] Meunier A., Jouannot O., Fulcrand R., Fanget I., Bretou M., Karatekin E., Arbault S., Guille M., Darchen F., Lemaître F., Amatore C. (2011). Angew. Chem..

[cit9] Estrada-Leypon O., Moya A., Guimera A., Gabriel G., Agut M., Sanchez B., Borros S. (2015). Bioelectrochemistry.

[cit10] Hoffmann C., Gaietta G., Bünemann M., Adams S. R., Oberdorff-Maass S., Behr B., Vilardaga J.-P., Tsien R. Y., Ellisman M. H., Lohse M. J. (2005). Nat. Methods.

[cit11] Stolwijk J., Matrougui K., Renken C., Trebak M. (2014). Eur. J. Appl. Physiol..

[cit12] Giaever I., Keese C. R. (1991). Proc. Natl. Acad. Sci. U. S. A..

[cit13] Gooding J. J., Parker S. G., Lu Y., Gaus K. (2013). Langmuir.

[cit14] Juliano R. (2002). Annu. Rev. Pharmacol. Toxicol..

[cit15] Le Saux G., Magenau A., Gunaratnam K., Kilian K. A., Böcking T., Gooding J. J., Gaus K. (2011). Biophys. J..

[cit16] Cavalcanti-Adam E. A., Volberg T., Micoulet A., Kessler H., Geiger B., Spatz J. P. (2007). Biophys. J..

[cit17] Roe M., Lemasters J., Herman B. (1990). Cell Calcium.

[cit18] Liu B., Chen W., Evavold B. D., Zhu C. (2014). Cell.

[cit19] Prime K. L., Whitesides G. M. (1993). J. Am. Chem. Soc..

[cit20] Ruoslahti E. (1996). Annu. Rev. Cell Dev. Biol..

[cit21] Kilian K. A., Böcking T., Gaus K., Gal M., Gooding J. J. (2007). Biomaterials.

[cit22] Chockalingam M., Magenau A., Parker S. G., Parviz M., Vivekchand S., Gaus K., Gooding J. J. (2014). Langmuir.

[cit23] May K. M. L., Wang Y., Bachas L. G., Anderson K. W. (2004). Anal. Chem..

[cit24] Aguilar-Maldonado B., Gómez-Viquez L., García L. A., Del Angel R. M., Arias-Montaño J. A., Guerrero-Hernández A. N. (2003). Cell. Signal..

[cit25] Giaever I., Keese C. R. (1993). Proc. Natl. Acad. Sci. U. S. A..

[cit26] Wegener J., Hakvoort A., Galla H.-J. (2000). Brain Res..

[cit27] Moy A. B., Winter M., Kamath A., Blackwell K., Reyes G., Giaever I., Keese C., Shasby D. (2000). Am. J. Physiol. Lung Cell. Mol. Physiol..

[cit28] Alexander J. S., Elrod J. W. (2002). J. Anat.

[cit29] Mrksich M. (2002). Curr. Opin. Chem. Biol..

[cit30] Wang Y., Mattson M. P., Furukawa K. (2002). J. Neurochem..

[cit31] Ribeiro C. M. P., Reece J., Putney J. W. (1997). J. Biol. Chem..

[cit32] Janmey P. A. (1994). Annu. Rev. Physiol..

