# Supplementary material for: Simultaneous impedance spectroscopy and fluorescence microscopy for the real-time monitoring of the response of cells to drugs
Source: Chem Sci. 2017 Jan 3;8(3):1831–40. doi: 10.1039/c6sc05159f (PMC5396555; doi:10.1039/c6sc05159f)
Supplement: Supplementary file 1 [file SC-008-C6SC05159F-s001.pdf]

Electronic Supplementary Material (ESI) for Chemical Science.
This journal is © The Royal Society of Chemistry 2017

Electronic supplementary information (ESI)

Simultaneous Impedance Spectroscopy and Fluorescence Microscopy for the Real-Time Monitoring of the Response of Cells to Drugs

Maryam Parviz, Katharina Gaus, J. Justin Gooding*

1. Simultaneous live microscopy and real-time impedance measurement

Pictures taken from the simultaneous optical/electrochemical setup and the custom-made electrochemical/optical chamber are shown in Fig S1.

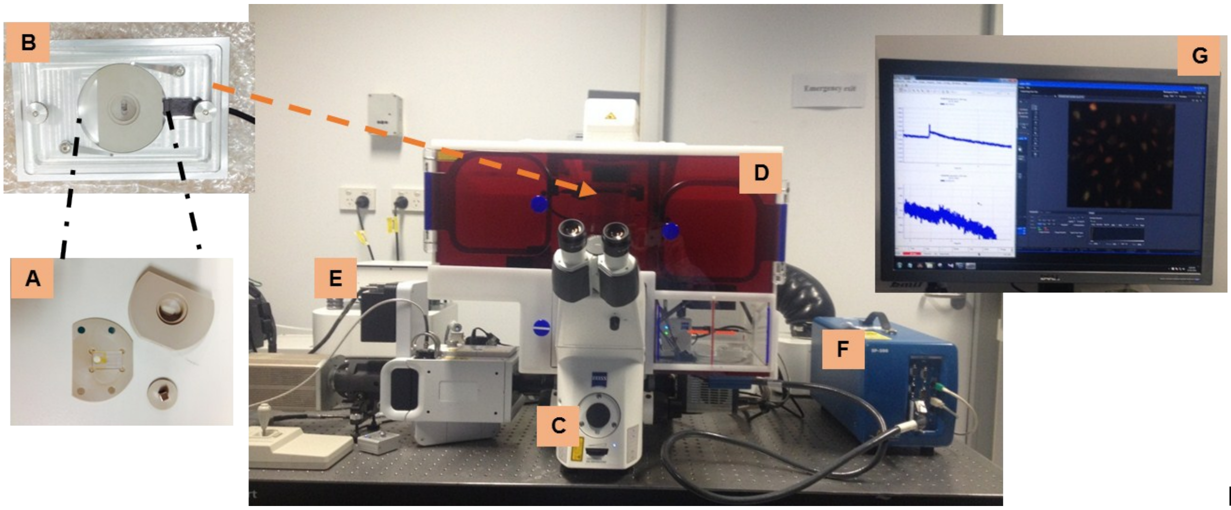

Fig. S1 Components of simultaneous live microscopy and impedance spectroscopy measurements setup; (A) the custom-made chamber for interdigitated ITO electrodes, (B) the microscope stage, (C) Inverted microscope, (D) the microscope cage, (E) Temperature and CO_2 controller units, (F) potentiostat and (G) a personal computer.

2. Finding the most sensitive frequency

During simultaneous measurements, the impedance values at 12 frequencies between 40000-400 Hz (10 mV) were recorded over time. The amplitude of current passed through the cells was held in the nanoampere (nA) range, which created a negligible electrical stimulation to cells during measurement. The real-time histamine-induced impedance alterations at the frequencies of 4000 Hz (black solid line) and 40000 Hz (grey dashed line) were compared together. These frequencies are among the most studied frequencies in the literature and were first time used by Giaever group.[1] We found that the change of histamine-induced impedance value was maximized at 4000 Hz (Fig. S2). This is in agreement with studies by other groups on searching the frequency at which the cell layer display the greatest histamine-induced impedance alteration.[2-4] This optimal frequency has been selected for further characterizations,

except the modeling studies which involve the impedance reading at the whole frequency range.

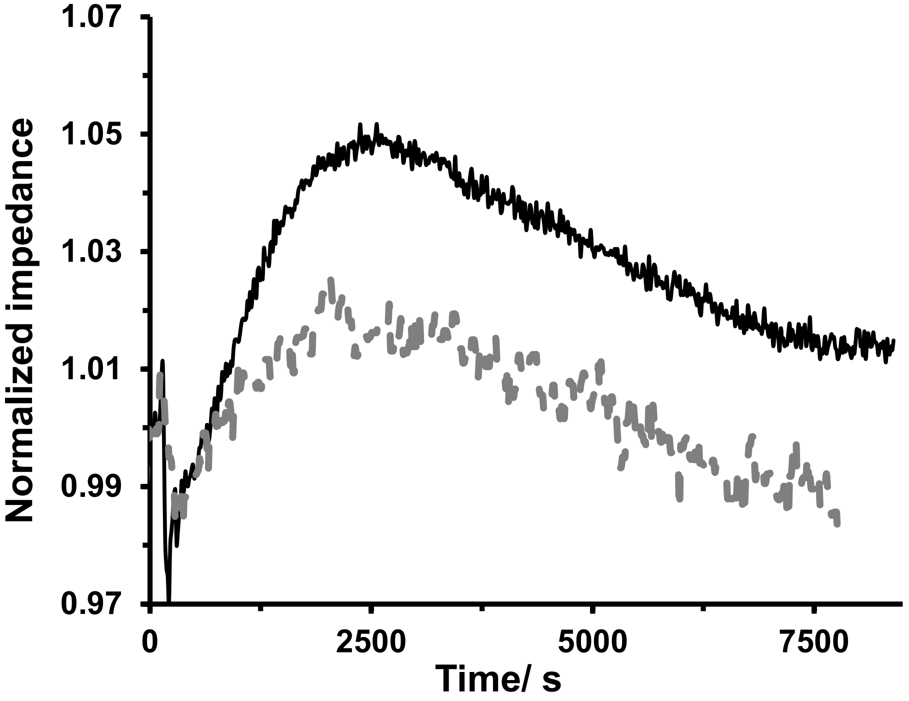

Fig. S2 The normalized impedance changes were calculated by dividing the value of the histamine-induced impedance of the cell-covered electrode to the impedance value of cells before histamine stimulation at frequencies of 4000 Hz (black solid line) and 40000 Hz (gray dashed line). The impedance measurement at the frequency of 4000 Hz showed the broadest range of relative change.

3. Assessing the potential effects of fura-2 imaging on cells

In order to ensure that fura-2 loading and excitation process have no interruption in cell bahaviour, phase contrast images and impedance signals from cells on interdigitated ITO electrodes were recorded without labeling cells with fluorophore dyes (Fig. S4). The similar trend of impedance result of this experiment compared with the impedance data recorded during simultaneous Ca^{2+} flux and impedance measurements, Fig. 2 A, for instance, indicated that loading cells with fura-2 and then exciting them for the time of Ca^{2+} signal measurement (in this study, not more than 20 min) had no observable effect on cells behavior upon histamine addition.

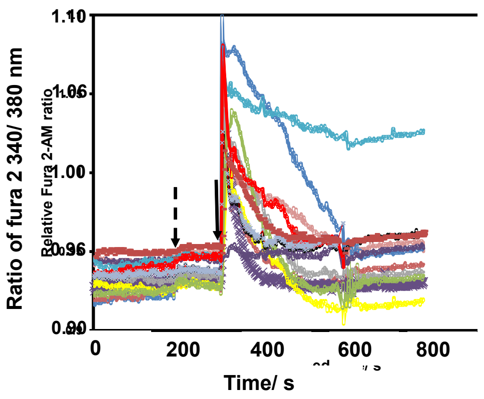

Fig. S3 Each curve presents the change for one single cell. The ratiometric values of many cells in the field of view are shown. These data were used to calculate the average change in ratiometric value. The arrows indicate the time of addition of Hanks' balanced salt solution (dashed arrow) and histamine (solid arrow).

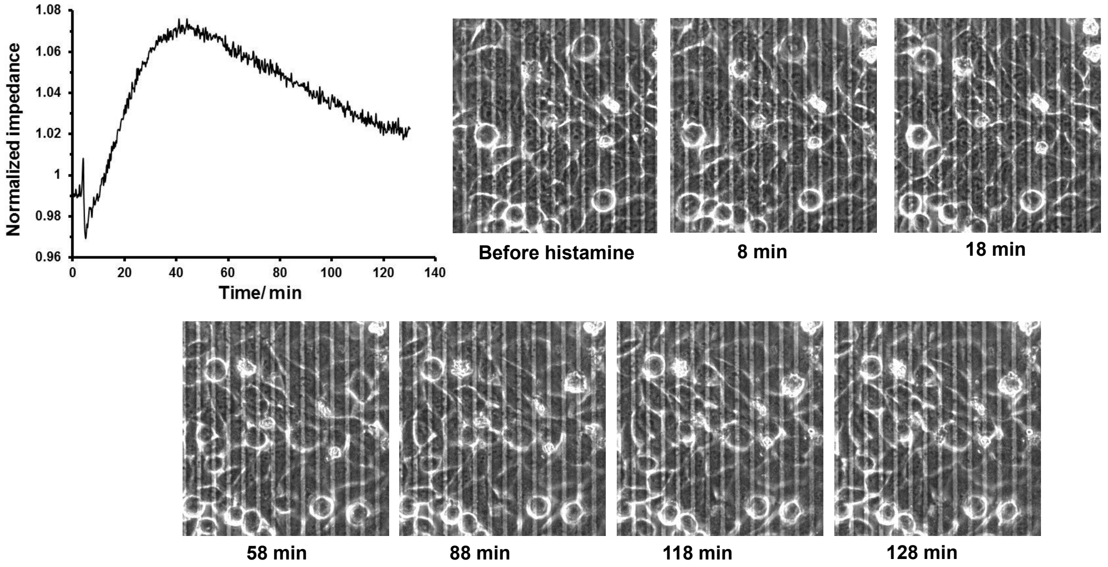

Fig. S4 The impedance response of cells on the surface with 31 nm RGD spacing with corresponding phase contrast images recorded simultaneously. The impedance response is in consistence trend with impedance response of fura-2-loaded cells as shown in Fig 2A, for instance. These results on long term impedance measurements indicate that the fura-2 loading and excitation process for the time of experiment have no observable influenced on cells behavior.

4. Modeling the impedance of the cell layer

Fig. S5 illustrates the frequency resolved impedance spectra of cell-free and cell-covered interdigitated ITO before and 25 min after histamine addition

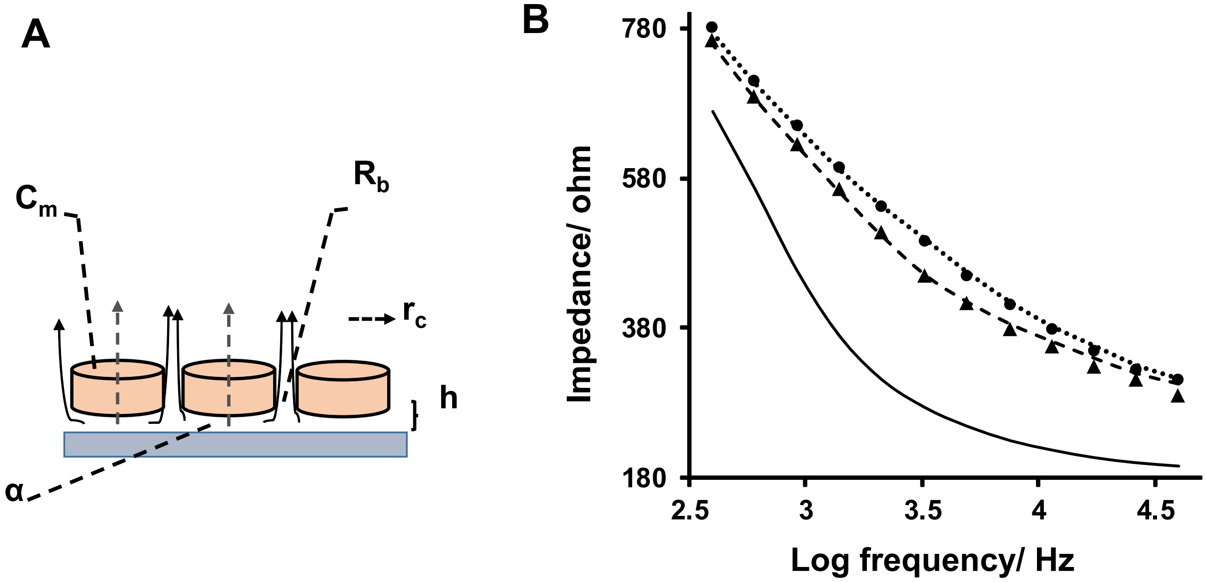

Fig. S5 (A) A mathematical model developed by the Giaever group was used to fit the frequency resolved impedance data to calculate the values of α, representing the contribution of cell-surface connection, R_b, showing the role of cell-cell strength in defining the impedance value, and $C_{m,}$ the cell membrane capacitance. The model also can be used to extract the morphological information of cells. r_c represents the average cell radius and h is the average distance between the ventral cell membrane and the surface. (B) An example of frequency resolved impedance data of a cell-free interdigitated ITO and the experimental (lines) and fitted (scattered dots) data of the cell-covered electrode before (dashed line and scattered triangles) and 25 min (dotted line and scattered circles) after histamine addition.

5. Cell imaging on plain ITO

It is worth noting that the current resolution of phase contrast images can be enhanced by improving the design and fabrication methods of interdigitated ITO electrodes, e.g. decreasing the total thickness of the underlying quartz substrate. Fig. S6 shows an optical image of cells on plain ITO surface. This confirms that the low-quality of the phase contrast images is not an inherent issue of ITO surfaces.

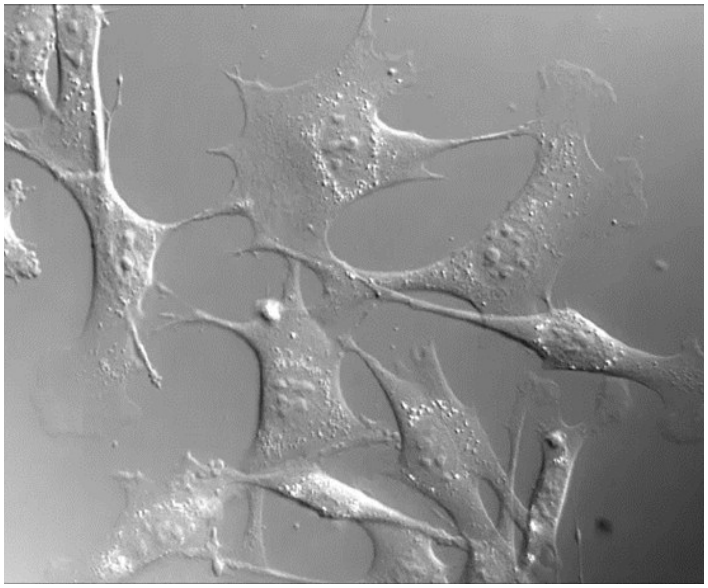

Fig. S6 An example of the obtained high quality image of cells cultured on ITO surfaces.

6. Surface modification characterization

The core level XPS spectra for carbon, nitrogen and phosphorous on ITO surface after each step of modification including (A) assembly of PHDA molecules, (B) activation of COOH groups using EDC and NHS, (C) coupling to 1-aminohexa(ethylene oxide) moieties, (D) activation of NH_2 groups using DSC and DMAP and (E) attachment to GRGDS ligands are shown in Fig. S7.

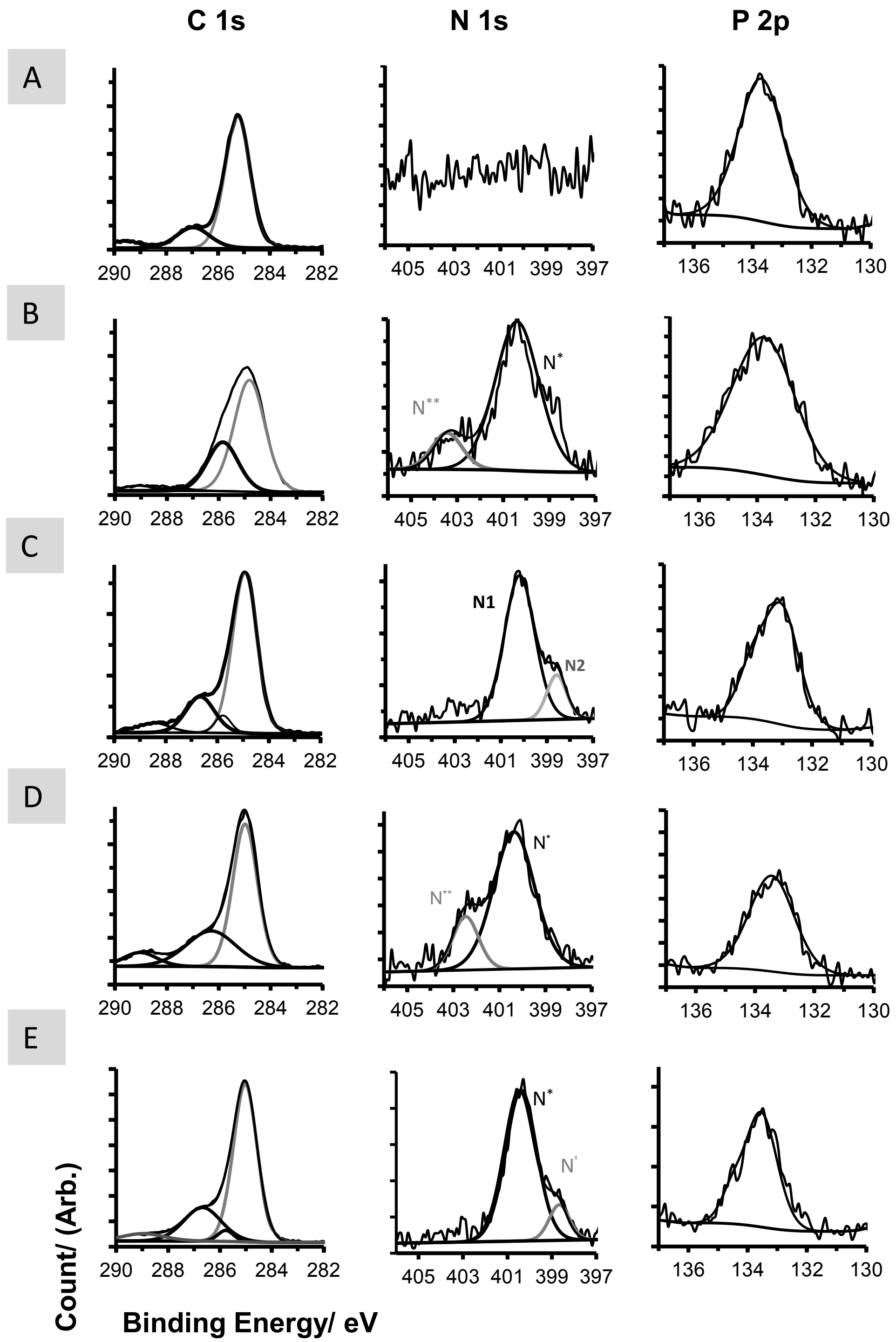

Fig. S7 C 1s, N 1s and P 2p XPS spectra of ITO surfaces after each step of modification: (A) carboxyl terminated PHDA SAM formation, (B) carboxyl group activation using EDC/NHS (C) 1-aminohexa (ethylene oxide) attachment (D) hydroxyl group activation using DSC/DMAP, and (E) GRGDS ligands attachment. XPS results indicate the modification of ITO surface with respective molecules.

The P 2p core level regions of XPS analyses show a main peak centered at around ~133.5 eV assigned to the phosphonate group. The C 1s high-resolution spectra were fitted with several peaks with the main peak centered at ~284.5 eV, which corresponds to aliphatic carbon bonded carbon (C−C). The fitted peaks at ~286.5 eV in C 1s regions are characteristic of the carbon atoms bound to oxygen, which shows an increase in its size in the case of the modifying surface using ethylene oxide rich molecules. The high binding energy peak at around 289.5 eV assigned to the carboxyl functional groups. The emergence of new peaks at the binding energy related to the carbonyl group, which is 287.5–288.1 eV,[5] were observed in the C 1s core level region of surfaces after carboxyl activation, ethylene oxide molecules attachment, DSC activation, and GRGDS coupling. The N 1s region shows the absence of nitrogen peak after the first step of modification using PHDA layer. However, after activation with EDC and NHS, the N 1s signal was detectable at ~402 eV in the survey scan assigned to an active succinimide ester-terminated SAM, and at ~399.8 eV attributed to *N*-acylurea intermediates. After putting ethylene oxide molecules on the surface, the high-resolution N 1s scan was deconvoluted with fitting to two functions: a peak at ~399 eV, attributed to *N*-acylurea intermediates and a peak at 400.1 eV correlated to the formation of an amide bond, which show successful nucleophilic binding of the 1-aminohexa(ethylene oxide) molecules. In the N 1s narrow scans of the DSC-activated ITO surface (D), the spectra were fitted with two peaks centered at ~400 eV and ~402 eV attributed to amide bound and the succinimidyl esters, respectively. The GRGDS modified surface was fitted with the similar peaks as 1-aminohexa (ethylene oxide) in the N 1s region; however, an increase in the peak at 400 eV, originating from amide groups within the peptide on the surface further supports that cell adhesive GRGDS peptides were attached to the activated succinimidyl ester terminated SAM via formation of amide bonds.

The results of XPS analyses were used to estimate the average RGD spacing of the prepared surfaces using a strategy that we have reported previously.[6] Briefly, the coupling efficiency of the connecting of 1-aminohexa(ethylene oxide) molecules to SAM and GRGDS to activated hydroxyl groups were calculated from XPS data to be approximately 72% and 42%, respectively. Thus, the overall efficiency was calculated to be 34%. The surface coverage of organophosphonate has been reported to be around

4 × 10^{14} molecules/cm^2 by Reven group.[7] Therefore, the estimated average spacing between adhesive ligands for the surface modified with 100% 1-aminohexa(ethylene oxide) molecules was calculated to be 1 nm. Subsequently, the average RGD spacing for surfaces modified with 1:10^3, 1:10^6 or 1:10^9 ratios of 1- aminohexa(ethylene oxide) monomethyl ether to 1-aminohexa(ethylene oxide) were estimated to be 31 nm, 970 nm, and 31000 nm, respectively.

To further characterize the modified surfaces, cyclic voltammograms before and after modification of an interdigitated ITO electrode with self-assembled PHDA and GRGDS were recorded. The measurements were performed in 1 mM ferricyanide at a scan rate of 100 mV s^{-1} (Fig. S8).The self-assembled layer induced a suppression in current compared with bare interdigitated ITO, providing an evidence of a well-formed PHDA layer on the surface. The interdigitated ITO surfaces were then modified with GRGDS peptides via activated hydroxyl groups of 1-aminohexa(ethylene oxide) molecules on the surface. The modification with GRGDS peptides suppressed the cyclic voltammogram obtained after PHDA SAM modification.

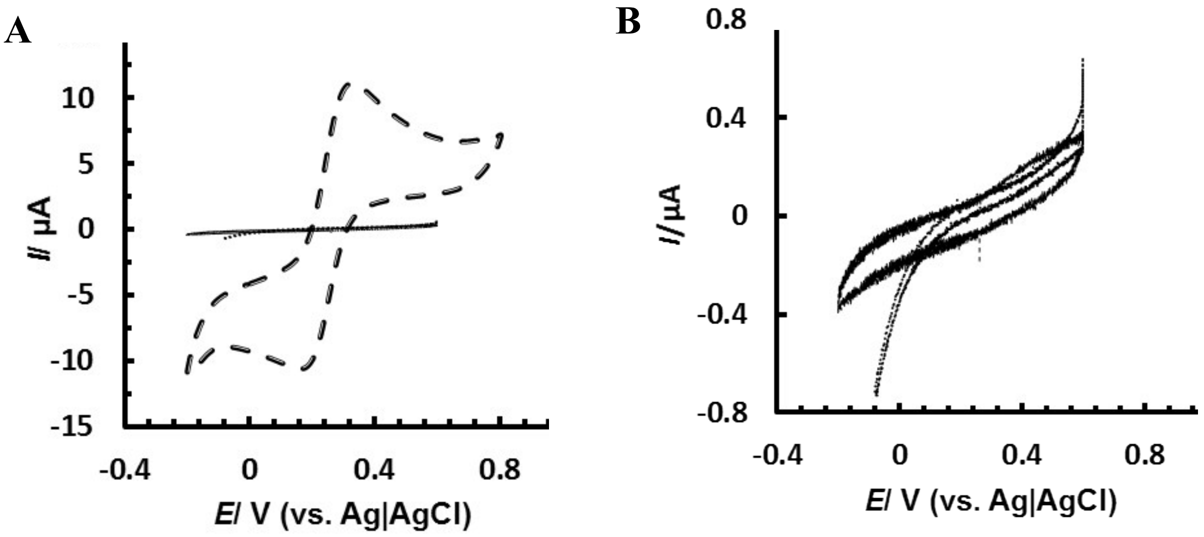

Fig. S8 (A) Cyclic voltammograms recorded in 1 mM ($[Fe(CN)_6]^{-3/-4}$) redox couple containing 0.1 M potassium chloride at the scan rate of 0.1 Vs^{-1} for bare interdigitated ITO (dashed line), PHDA SAM- (solid line), and GRGDS (dotted line) modified surfaces. (B) Magnified presentation of cyclic voltammograms of PHDA SAM-modified ITO (solid line) and GRGDS modified ITO surface (dotted line). The reduction in current is an indication of the surface passivation after each step of modification.

7. Modeled impedance data on the surfaces with different RGD spacing

The relative contribution of change in α, representing the cell-surface adhesion and R_b, cell-cell resistance, in altering histamine-induced impedance signal on surfaces with different RGD spacing is shown in Fig. S9. The relative changes were calculated with respect to the relevant values for each parameter before histamine addition. These values are calculated by subjecting the frequency resolved impedance data at 3 and 25 min after histamine addition to the mathematical modeling as was discussed in section 5. According to Fig. 6A, after 3 min of histamine stimulation, cells on all of the surfaces exhibited a decrease in their histamine-induced impedance alteration. The impedance value of the cell layers on different surfaces after 25 min of histamine addition were higher than the baseline. Therefore, the impedance results recorded at these time points were summarized here.

The results of modeling demonstrated that the cell-cell and cell-substrate adhesions decline more (t =3 min) and, show greater change toward reaching the maximum value (t =25 min) on the surface with RGD spacing in the range of micrometers compared with cells on the surfaces with average 1 nm or 31 nm RGD spatial distribution. It is believed that this is mainly due to the fact that cells on surfaces with RGD spacing of micrometers are less strongly bonded to the surface and may be less close to their full contractile and spreading potential. This finding is in agreement with our previous study[8] where endothelial cells on a silicon surface with average 1,380 nm or 43,700 nm RGD spacing displayed a higher migratory ability than cells on silicon surfaces with average 1.4 nm or 44 nm RGD spacing.

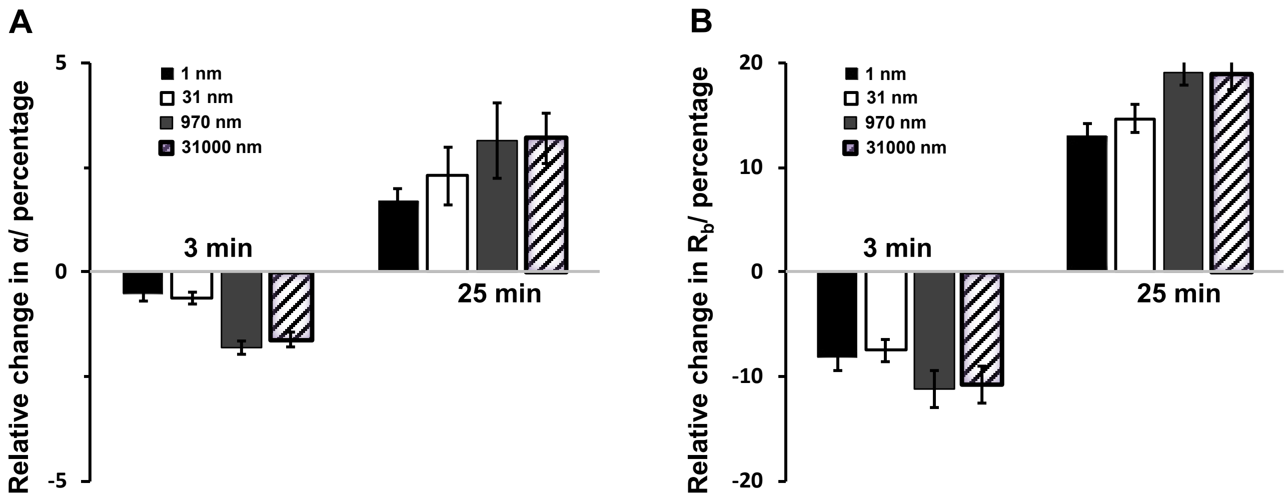

Fig. S9 Calculated relative changes in (A) α, representing the cell-surface adhesion and (B) R_b, cell-cell resistances, after 3 min and 30 min. The relative values are obtained by calculating the percentage change of cell-cell or cell-surface resistances compared with the respective values before histamine addition on each surface.

References

[1] J. Wegener, C. R. Keese, I. Giaever, *Exp Cell Res* **2000**, *259*, 158-166.

[2] A. B. Moy, M. Winter, A. Kamath, K. Blackwell, G. Reyes, I. Giaever, C. Keese, D. Shasby, *Am J Physiol Lung Cell Mol Physiol* **2000**, *278*, L888-L898.

[3] I. Giaever, C. R. Keese, *Proceedings of the National Academy of Sciences* **1991**, *88*, 7896-7900.

[4] I. Giaever, C. Keese, *Proc. Natl. Acad. Sci. USA* **1984**, *81*, 3761-3764.

[5] S. Biniak, G. Szymański, J. Siedlewski, A. Świątkowski, *Carbon* **1997**, *35*, 1799-1810.

[6] M. Chockalingam, A. Magenau, S. G. Parker, M. Parviz, S. Vivekchand, K. Gaus, J. J. Gooding, *Langmuir* **2014**, *30*, 8509-8515.

[7] S. Pawsey, K. Yach, L. Reven, *Langmuir* **2002**, *18*, 5205-5212.

[8] G. Le Saux, A. Magenau, K. Gunaratnam, K. A. Kilian, T. Böcking, J. J. Gooding, K. Gaus, *Biophys. J.* **2011**, *101*, 764-773.
